# Thrombin contributes to the injury development and neurological deficit after acute subdural hemorrhage in rats only in collaboration with additional blood-derived factors

**DOI:** 10.1186/s12868-018-0481-5

**Published:** 2018-12-27

**Authors:** Tobias J. Krämer, Wasim Sakas, Daniel Jussen, Harald Krenzlin, Oliver Kempski, Beat Alessandri

**Affiliations:** 1 Institute for Neurosurgical Pathophysiology, University Medical Center of the Johannes Gutenberg-University Mainz, Langenbeckstrasse 1, 55131 Mainz, Germany; 2 Department of Neurosurgery, HELIOS Dr. Horst Schmidt Kliniken, Wiesbaden, Germany; 3 Department of Neurosurgery, University Medical Center of the Johannes Gutenberg-University Mainz, Mainz, Germany

**Keywords:** Acute subdural hemorrhage, Thrombin, Neurotoxicity, Argatroban, PAR1, SCH79797

## Abstract

**Background:**

Acute subdural hemorrhage (ASDH) is a severe consequence of traumatic brain injury. The occurrence of subdural blood increases the lethality of these patients independent of the amount of blood or elevated intracranial pressure. Thrombin is one of the potential harmful blood components. Possible harmful effects of thrombin are mediated via the Protease-activated-receptor-1 (PAR1) and thus, translating the acute Thrombin release after ASDH into cell loss. The objectives of the present study were twofold, namely to examine (1) the impact of direct thrombin inhibition in the acute phase after hemorrhage on the long-term histological and functional deficits and (2) the early inhibition of PAR1 activation by thrombin with the selective antagonist SCH79797 on lesion volume at 14 days after ASDH. The effects of thrombin on the lesion size were investigated in two separate experiments via (1) direct thrombin inhibition in the subdural infused blood (Argatroban 600 µg) as well as by (2) intraventricular injection of the PAR-1 antagonist SCH79797 (1 µg or 5 µg). Lesion volume and behavior deficits using a neurological deficit score and a motor function test (beam balance test) were analyzed as outcome parameters at 14 days after injury.

**Results:**

59 Male Sprague–Dawley rats received a subdural infusion of 300 µl autologous blood or sham operation. Lesion volume at 14 days after ASDH tended to be smaller in the Argatroban-treated group when compared to the vehicle group (8.1 ± 1.1 vs. 10.1 ± 2.3 mm^2^, n.s.). Motor deficits in the beam balance test were not significantly less severe in the Argatroban-treated group. Animals treated with SCH79797 also showed a trend towards dose-dependent decreased lesion volume in comparison to the vehicle-treated group (1 μg: 4.3 ± 0.7 mm^3^; 5 μg: 3.8 ± 1.1 mm^3^; vehicle: 6.5 ± 2.0 mm^3^, n.s).

**Conclusions:**

Thrombin inhibition in the subdural blood and local cerebral blockade of PAR-1 cause a tendency towards reduced lesion volume or functional recovery. All results show a trend in favor of the acute treatment on the outcome parameters. Our results suggests that thrombin could be an important blood-derived factor during acute subdural hemorrhage that translates its deleterious effects in concert with other blood-induced factors.

## Background

The acute subdural hematoma (ASDH) represents a severe complication in patients suffering from traumatic brain injury (TBI). Death or poor neurological discharge status is still common after ASDH, and the rates of poor outcome did not improve in the last decades [1]. Lifetime costs for survivors in 2010 were 38 million US$ per year per one million inhabitants and are expected to reach 50 million US$ in 2020 [2]. The current medical management of TBI is primarily symptomatic due to a lack of pharmacological therapies to improve neurological outcome.

Unaccountable large lesion volumes have been reported in autopsies of TBI patients, which were not corresponding to the initial trauma. It was assumed that the cranial-perfusion-pressure (CPP) was depressed by the raising intracranial-pressure (ICP) after traumatic ASDH [3]. The first rodent model for ASDH was developed by Miller in 1990 to investigate this issue [4]. It was hypothesized that the solitary presence, and not the amount of blood, could be responsible for the severe expansion of the secondary damage [4]. This notion was supported by substituting subdural blood by silicone gel infusion which increased cerebral blood flow after cerebral “hematoma” compared to autologous blood [5]. These data suggest that tissue pressure and vasoactive substances are components of the immediate reduction in blood flow following intracranial hemorrhage. Our group objectified this thesis for the ASDH by comparing the subdural infusion of blood to an inert volume substance, paraffin oil, with simultaneous ICP, CPP and cerebral blood flow (CBF) measurement and testing of the functional outcome [6, 7]. These results showed that the development of secondary brain damage after ASDH is not just a consequence of raising ICP, but of additional pathological mechanisms, which become initiated by blood constituents itself. Different lesion volumes 96 h after ASDH with blood or paraffin oil in rats highlight the injuring effect of blood in direct contact to brain parenchyma [7]. Blood triggered to a much greater extent than paraffin oil cerebral edema, reduction of glucose metabolism and tissue death up to 48 h after ASDH induction. In the acute phase after ASDH induction, ICP increase and CBF drop were independent of the infused substance [6]. Several attempts to identify reproducible harmful mechanisms mediated by the contact of blood with brain tissue in different animal models could not provide clarification [8, 9]. Besides the volume of subdural blood, it is still unknown which components of the blood contribute to increasing ICP, decreasing tissue oxygen concentration, the rise of glutamate and lactate in company with extensive edema, cell death and larger lesions [7, 9].

A potentially harmful blood-derived factor might be thrombin. Mainly derived from the liver, thrombin permeates the brain parenchyma after bypassing the blood–brain–barrier (BBB) following e.g., intracerebral or subdural hemorrhage and in mild TBI [10]. A small portion of thrombin and prothrombin is expressed in neural cells [11], and this is associated with chronic neurodegenerative diseases like Alzheimer [12], Parkinson [13] and Multiple Sclerosis [14]. The neurotoxic effects of thrombin become exaggerated in inflammable, ischemic or hypoxic conditions [14]. Thrombin causes synaptic dysfunction [15], vascular disruption and enhances the inflammatory response and neuronal damage via PAR1 activation [16, 17]. Systemic thrombin inhibition with the direct thrombin inhibitor Argatroban already improved early brain injury and neurological outcome after subarachnoid hemorrhage in rats [18]. Argatroban derives from the amino acid arginine and binds to the active catalytic binding site of thrombin. Originally it is approved to prevent clot formation in patients at risk for heparin-induced thrombocytopenia [19]. It is a strong, low molecular weight and monovalent direct thrombin inhibitor. The activity of Argatroban is based on inhibition of all thrombin effects, including fibrin formation, as well as platelet activation and aggregation and binding to PARs. Binding takes place on dissolved as well as on fibrin-bound thrombin [20, 21]. Direct thrombin blocking is also possible with Bivalirudin, which is a standard anticoagulant during a percutaneous coronary intervention (PCI) or transcatheter aortic valve implantation (TAVI). The distinct shorter halftime of Bivalirudin over Argatroban, perfect for the short interventions mentioned, was an argument in favor of using Argatroban for our purposes. The neuroprotective effects after systemic administration in ischemic conditions are most likely explained by the systemic anticoagulation of Argatroban [18, 22]. Our present study refers to the acute local inhibition of thrombin and PAR1 as well as their effects on the longer-term outcome. Once released thrombin activates the PAR1 on neurons, astrocytes, oligodendrocytes and microglia [11, 17, 23]. Therefore, a second arm (2) was implemented in the study, in which the effects of the PAR1 receptor with the specific antagonist SCH79797 are examined separately from the thrombin effect. Activation of PAR1, a G-protein coupled receptor, is caused by cleaving its extracellular N-terminal domain by e.g. thrombin [24, 25] and induces changes in glutamate uptake of the cell [16, 26]. Thrombin serves via PAR1 as an extracellular “death signal” to activate intracellular protease pathways which lead to apoptotic cell death [27]. Thrombin induced membrane lipid peroxidation is also mediated in part through PAR1 and results in neuronal cell loss in various CNS degenerative and traumatic pathologies [28]. Furthermore, there is a transactivation signaling network between PAR1 and epidermal growth factor (EGFR) that is mediated by vascular endothelial growth factor (VEGFR-2) which results in synergistic MAP kinase phosphatase-1 (MKP-1) induction with consecutively increased inflammatory response and apoptosis [29]. Among other factors, apoptosis is mediated via phosphatidylinositol 3-kinase (PI3 K), a key signaling enzyme implicated in cell survival [30]. SCH79797 dependent inhibition of PAR-1 preserves against harmful effects induced by inflammatory activation in neural cells via PI3 K/Akt pathway. SCH79797 protected dose dependent against LPS-induced microglial activation in vitro. Neuronal cell death via up-regulation of Akt-mediated inflammation was significantly reduced after SCH79797 treatment [31]. The selective PAR1-antagonist SCH79797 has already proven to induce protective effects 24 h after surgical trauma [32], 48 h after ischemia and reperfusion [33] and to reverse trauma-induced amnesia in mice 1 day after trauma [34].

We hypothesize that the acute release of thrombin from the subdural hematoma could be one of the harmful blood constituents, which trigger long-term lesion enlargement through early activation of PAR1. In the first part of this trial (1) the direct thrombin inhibitor Argatroban, which prevents the acute release of thrombin from the subdural infused blood is employed and in a second study (2) the PAR-1 antagonist SCH79797 which inhibits most of the cellular effects of thrombin [35] is installed as a local application in an ASDH model in rats.

## Results

### Acute monitoring of physiological parameters

In both arms of the study, two arterial blood samples were taken in every animal to document and maintain physiological parameters during artificial ventilation. One probe was taken during the baseline adjustment and one at the end of the acute observational period (− 10 to + 60 min). All parameters of the blood samples were in a physiological range and did not differ between the groups. Middle arterial blood pressure (MAP) did not differ between the groups during the 10-min baseline monitoring. In the sixth minute after the start of the subdural infusion MAP raised to the maximum in all ASDH groups for approximately 30 min. Thereafter MAP dropped back to baseline levels at the end of the 60-min acute observation period. No difference in the MAP between the groups turned up in the recordings. The MAP, pH, the partial pressure of pCO_2_, pO_2_, standard bicarbonate, standard base excess, hemoglobin, hematocrit, glucose, and lactate are presented in Table 1. Taken the MAP and blood samples data together there is no evidence of anesthesia- or drug-related effects which influenced blood circulation and all other endpoints of the study.

**Table 1:** Blood-gas-analysis and MAP: in both arms of the study (thrombin blockage with argatroban and PAR1-antagonism with SCH79797) two arterial blood samples were taken

Group	MAP (mmHg)	pH	pCO_2_ (mmHg)	pO_2_ (mmHg)	SHCO_3_ (mmol/l)	SBE (mmol/l)	Hb. (g/dl)	Hkt. (%)	Gluc. (mg/dl)	Lactate (mmol/l)
*Argatroban: baseline calibration*										
Sham	65.1 ± 4.2	7.38 ± 0.02	46.1 ± 2.4	149.0 ± 8.9	261 ± 0.5	2.5 ± 0.6	14.3 ± 0.2	43.8 ± 0.7	242.8 ± 20.0	1.9 ± 0.2
Vehicle	66.3 ± 3.8	7.40 ± 0.02	45.4 ± 2.3	143.0 ± 7.7	26.9 ± 0.6	3.0 ± 0.6	14.1 ± 0.3	43.4 ± 0.8	243.4 ± 22.7	1.9 ± 0.3
Argatroban	63.5 ± 5.7	7.40 ± 0.02	40.5 ± 2.2	134.8 ± 9.3	25.5 ± 1.0	1.2 ± 1.2	14.0 ± 0.5	43.0 ± 1.6	261.3 ± 18.3	1.8 ± 0.3
*Argatroban: end of procedure*										
Sham	68.3 ± 4.1	7.29 ± 0.02	48.9 ± 4.9	112.3 ± 7.3	23.0 ± 1.8	0.9 ± 2.4	13.1 ± 1.0	40.4 ± 3.1	212.7 ± 61.3	1.4 ± 0.3
Vehicle	69.0 ± 3.3	7.35 ± 0.06	41.6 ± 7.1	123.3 ± 2.6	21.6 ± 1.4	3.3 ± 16	13.0 ± 0.2	40.1 ± 1.6	224.0 ± 20.4	1.2 ± 0.3
Argatroban	70.1 ± 4.6	7.33 ± 0.3	48.0 ± 4.8	124.7 ± 9.8	23.5 ± 1.4	0.5 ± 1.7	13.8 ± 0.4	42.2 ± 1.1	293.8 ± 28.4	1.7 ± 0.4
*SCH*-*79797: baseline calibration*										
Sham	85.0 ± 1.0	7.38 ± 0.02	42.0 ± 3.0	137.8 ± 8.7	25.2 ± 1.0	0.6 ± 1.1	13.8 ± 0.4	42.6 ± 1.3	296.3 ± 15.4	1.8 ± 0.2
Vehicle	77.7 ± 0.8	7.40 ± 0.02	43.4 ± 2.5	130.8 ± 11.2	26.9 ± 0.5	2.2 ± 0.8	13.5 ± 0.4	41.4 ± 1.2	258.4 ± 14.8	1.7 ± 0.4
SCH-1	76.9 ± 1.0	7.41 ± 0.01	37.7 ± 1.1	120.6 ± 8.1	25.1 ± 0.5	1.1 ± 0.5	12.3 ± 0.3	37.2 ± 1.1	306.8 ± 16.6	1.24 ± 0.16
SCH-5	79.9 ± 2.2	7.42 ± 0.01	39.0 ± 0.7	125.7 ± 7.9	26.5 ± 0.4	2.7 ± 0.6	12.8 ± 0.6	39.6 ± 2.0	301.2 ± 19.3	1.71 ± 0.3
*SCH*-*79797: end of procedure*										
Sham	81.7 ± 1.8	7.414 ± 0.01	40.7 ± 109	129.7 ± 5.4	26.2 ± 0.6	2.0 ± 0.5	14.3 ± 0.3	43.8 ± 1.0	291.3 ± 12.7	1.6 ± 0.2
Vehicle	75.1 ± 1.5	7.4 ± 0.1	41.1 ± 1.4	147.8 ± 3.7	25.7 ± 0.7	1.6 ± 0.7	13.1 ± 0.5	40.5 ± 1.4	250.0 ± 8.0	1.3 ± 0.3
SCH-1	78.2 ± 1.9	7.42 ± 0.01	36.8 ± 0.6	130.2 ± 4.7	24.6 ± 0.5	1.4 ± 0.4	12.7 ± 0.2	37.2 ± 1.1	296.8 ± 15.6	14 ± 0.1
SCH-5	76.9 ± 2.0	7.41 ± 0.02	37.9 ± 1.3	123.0 ± 6.6	25.3 ± 0.5	1.8 ± 0.5	12.3 ± 0.4	37.6 ± 1.4	265.4 ± 13.6	1.5 ± 0.2

One sample was taken during the CBF baseline measurement (− 10 to 0 min) and one at the end of the observational period (0–60 min after ASDH). All parameters were in a physiological range and did not differ between the groups. The MAP was monitored continuously. In the sixth minute after the start of the subdural infusion the MAP raised to a maximum in all ASDH groups. After that MAP dropped back within 30 min to baseline levels until the end of the observational period. Stable and physiological data in the blood-gas-analysis and comparable results in MAP suggest no differences between the groups due to anesthesia, ventilation or surgical procedures. All values mean ± SEM

### Acute monitoring of local cerebral blood flow

**Fig. 1:**
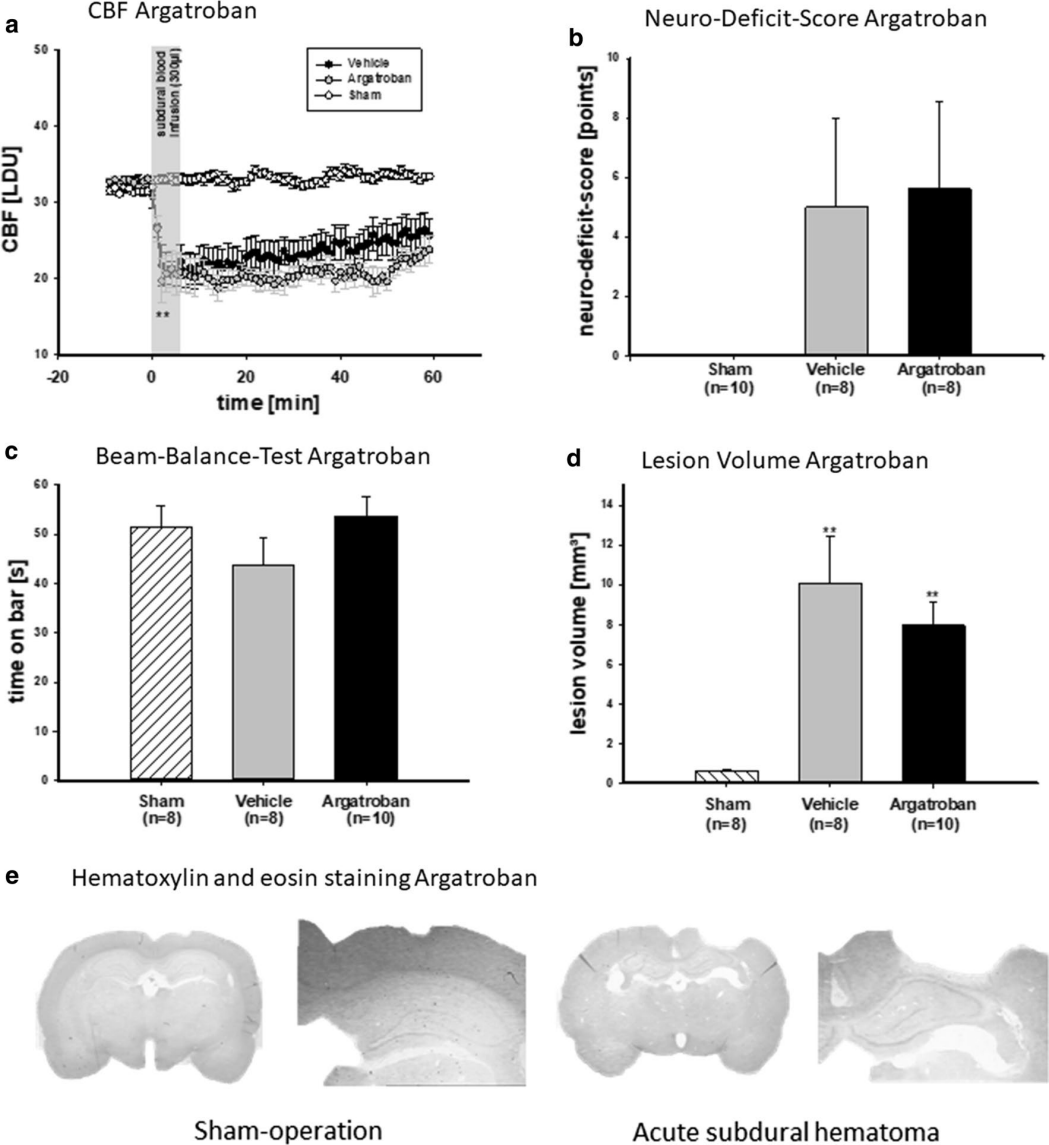
Argatroban treatment does not affect the cerebral blood flow (CBF, Graph A) during the acute phase of ASDH. Two minutes after start of subdural infusion CBF dropped in all rats with a ASDH significant beneath the sham-operated animals and stayed there until end of recording (second minute: sham 32.6 ± 0.9 LDU, vehicle 20.5 ± 1.3 LDU, Argatroban 18.7 ± 1.7 LDU; Argatroban vs. vehicle n.s., **Argatroban vs. sham *P* < 0.001; **vehicle vs. sham *P* < 0.001, mean ± SEM). The NDS could not distinguish between vehicle or Argatroban treatment. Graph B presents the mean of each group by three trials per animal. Similarly, treatment with Argatroban had no effect on beam balance (C) performance (maximum time on beam was 60 s). Bars present the mean of each group by three trials per animal (sham 51.6 ± 2.0 s, vehicle [saline] 43.7 ± 5.6 s, Argatroban 53.8 ± 4.1 s; vehicle vs. Argatroban *P* > 0.05). Direct inhibition of thrombin with Argatroban (600 µg Argatroban/300 µl subdural infused blood) leads to a discreetly improved functional result and a slight reduction in the histological lesion volume compared to vehicle solution. Graph D and E show lesion volumes induced by subdural infusion of 300 µl blood after 14 days (Argatroban vs. vehicle *P* > 0.05, vehicle vs. sham *P* < 0.001 and Argatroban vs. sham *P* < 0.001). Images show hematoxylin–eosin stained brain sections at 1.25- and 2.5 fold magnification

**Fig. 2:**
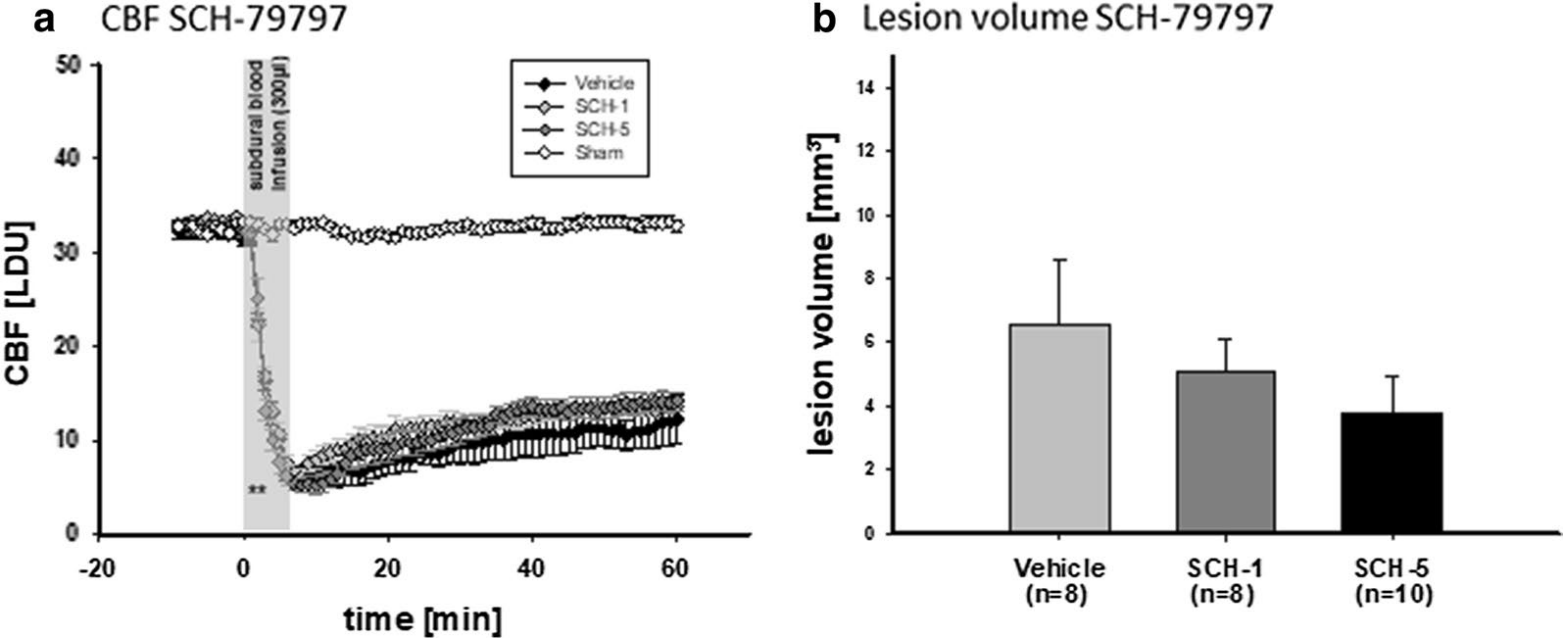
The local CBF was not affected by the PAR1-antagonist SCH 79797 after ASDH (left graph). CBF was stable during the baseline period (32.6 ± 0.5 LDU, mean ± SEM). In the second minute after ASDH induction CBF dropped down and was constantly significant underneath the sham-operated animals (**SCH-1 vs. sham *P* < 0.001; **SCH-5 vs. sham *P* < 0.001; **vehicle vs. sham *P* < 0.001). Local administration of the PAR1 antagonist SCH79797 results in a not significantly reduced neuronal damage 14 days after ASDH compared to vehicle treatment, similar to the Argatroban treatment (right graph)

Baseline measurements of cerebral blood flow (CBF) before induction of ASDH show stable conditions in the observed perilesional tissue (Figs. 1a, 2a). There was no difference between treatment groups during baseline (all p: n.s.). After infusion of 300 µl blood into the subdural space (2 min after start of the subdural infusion) there was a distinct difference of the ASDH groups to the sham-operated groups until the end of recording in the Argatroban (sham: 32.6 ± 0.8 LDU, vehicle: 20.6 ± 3.7 LDU, Argatroban: 18.7 ± 2.9 LDU; vehicle vs. sham: *P* < 0.001; Argatroban vs. sham: *P* < 0.01, Fig. 1a) and also in the SCH79797 treated animals (sham: 32.7 ± 0.5 LDU, vehicle: 12.3 ± 2.2 LDU, SCH1 µg: 13.6 ± 0.5 LDU, SCH5 µg: 13.9 ± 1.0 LDU; vehicle vs. sham: *P* < 0.001; SCH1 µg vs. sham: *P* < 0.001; SCH1 µg vs. sham: *P* < 0.001; Fig. 2a). In all non-sham groups, a significant decrease in CBF after subdural blood injection was documented. Previous operative trauma, mechanical ventilation, anesthesia or the treatment do not produce any fluctuations in the CBF.

### Body weight

All rats taken together had a mean body weight of 318.1 ± 5.4 g on the operation day. All groups presented a weight loss at post-operation day 1, reaching 276.5 ± 3.6 g and recovered to 348.3 ± 9.1 g within 14 days. After a post-operative loss, all animals exceeded their initial weight after 14 days, which is considered as an expression of good general conditions and adequate environmental requirements.

### Functional testing after Argatroban treatment

All animals in the Argatroban study-arm underwent functional testing on the 14th post-interventional day with no training on a Neuro Deficit Score (NDS) and a Beam Balance Test [36, 37]. From three runs the mean was established, and the examiner was blinded to the treatment. The NDS (ranging from zero: no impairment, to nine points: severe impairment) was 0 points for sham-operated animals, 5.0 ± 3.0 points for vehicle-treated animals and 5.6 ± 2.9 points for Argatroban-treated rats. Both ASDH groups had a significantly greater neurological deficit than the sham group (both to sham *P* < 0.05, Fig. 1b), but did not differ from each other. From three trials of the Beam Balance Test the meantime on the bar was established (Fig. 1c). Sham animals reached 51.6 ± 4.2 s on the beam before falling, vehicle treated remained on the beam for 43.7 ± 4.6 s whereas Argatroban-treated rats remained on the bar for 53.8 ± 4.1 s. A tendency to improved motor behavior was observed in the Argatroban group (n.s.).

### Lesion volume 14 days after ASDH

Thrombin blocking with Argatroban did not affect the lesion volume considerable. In the sham group, the surgery-induced damage amounted to 0.6 ± 0.1 mm^3^. The vehicle group had a lesion volume of 10.1 ± 2.3 mm^3^. Average tissue damage in the Argatroban group was 8.0 ± 1.2 mm^3^ (all p: n.s., Fig. 1d, e).

PAR1 blockage resulted in the following lesion volumes: Animals treated with vehicle had an average tissue loss of 6.5 ± 2.0 mm^3^, with one µg SCH-79797 lesion volume was 4.3 ± 0.7 mm^3^ and with five µg SCH-79797 the average damage was 3.8 ± 1.1 mm^3^ (all p: n.s., Fig. 2b).

The results of the volumetric lesion determination and the functional outcome both show a slight trend in favor of thrombin inhibition or PAR1 antagonism. No significant neuroprotective effect seems to be achievable with an acute and single treatment at this time alone. Nevertheless, there is a trend in favor of blockage of the thrombin/PAR1 axis for all measured parameters 14 days after ASDH. This suggests an involvement of thrombin as part of several other blood-derived factors, which contribute to the lesion growth after ASDH.

## Discussion

Thrombin could be one of the critical components, which modulate the neuronal damage after ASDH [14, 38]. A small proportion of cells in the central nervous system can release thrombin [39, 40]. It is known that gene expression for prothrombin and PAR1 is upregulated in ischemic brains [38, 41]. The blood of the subdural hematoma is also a “physiological” source of thrombin in the brain. This study presents no substantive protective propensities of Thrombin after ASDH via local direct thrombin and PAR1 inhibition. No statistical significance regarding the long-term consequences of neuronal damage and the functional outcome could be shown. Nevertheless, the results of this study play an essential role in the understanding of the development of secondary brain damage after ASDH. In two different standalone experimental settings, we demonstrate a tendency to decreased lesion volume and when tested, to functional recovery with blockage of thrombin or PAR1 compared to vehicle. These findings are primarily supportive to this hypotheses, but due to the lack of statistical significance in this study do not prove that thrombin is one of the key players in modulating secondary damage after ASDH, as seen in other CNS pathologies [39, 42]. However, there are other coagulation factors like activated protein C or the tissue-type plasminogen activator (t-PA), which play at least equal, if not more serious roles [43, 44].

Under physiological conditions, thrombin is unable to pass the BBB [45]. In our rat model, the source of the hematoma is not a ruptured blood vessel as it is seen in a traumatic injured human brain. Nevertheless, the BBB gets secondary impaired through pressure and ischemia. Therefore, thrombin from the systemic circulation can pass the BBB [46]. Thrombin itself harms the BBB [47], and also, the blood of the hematoma is also a “physiological” thrombin source. This work aimed foremost at the local thrombin in the hematoma between the dura mater and pia arachnoid, primary without direct contact to brain tissue. This fraction was specifically blocked with Argatroban before the subdural injection without affecting systemic coagulation (data not shown) to reduce the early adverse effects of thrombin in subdural accumulated blood [18]. The subdural and segregated localization of the hematoma as thrombin source might explain the weaker effects of thrombin-inhibition compared to trauma or subarachnoid bleeding [46].

Thrombin influences signal transduction in the brain predominantly via PAR1 [48, 49]. Although thrombin also uses PAR3 and PAR4 next to PAR1, it appears that modification and proliferation e.g., astrocyte activation, is mainly mediated via PAR1 [48]. PAR1 is localized on neurons, astrocytes, and endothelial cells and so its actions are intricate [49]. Activation of PAR1 with thrombin in cultured astrocytes contributes to excitotoxic neuronal injury by elevating glutamate release from glial cells [50]. PAR1 knockout mice showed less neurological deficits, endothelial barrier leakage, and neuronal degeneration compared to wildtype in a middle cerebral arterial occlusion model [51]. The data in this ischemia model of Rajput et al. does not refer to acute pharmacological treatment approaches but to PAR1 modification and to shorter survival periods when compared to our study. Contributing to this dataset, we present the results from our investigation, regarding a pharmacological intervention to block the PAR1 pathway in the acute phase of thrombin inundation into the surrounding brain tissue. We noticed a most likely dose-dependent effect to the lesion volume 14 days after ASDH with the PAR1 antagonist SCH79797. However, blocking thrombin or PAR1 at the time of formation of the subdural hematoma had no significant effects on lesion volume after 14 days, and therefore we cannot prove harmful neuronal effects by PAR1 or thrombin with local inhibition. But PAR1 inhibition by SCH79797 in equal doses has already shown positive effects in the early stage 24 h after surgical trauma in rats [32]. These therapeutic approaches seem only to relay neuronal loss for a few hours or days [18, 32–34], but do not prevent neuronal cell death for 14 days. It is likely that the spare amount of thrombin in the subdural hematoma, in contrast to the thrombin in the systemic circulation, is not sufficient to affect the damage or does not sufficiently permeate into surrounding parenchyma. This could lead to less neuroprotective effects of thrombin/PAR1-axis blockage in ASDH compared to hemorrhagic insults in rats [18, 22, 52]. As well as local Argatroban treatment, also a systemic administration could not induce neuroprotective effects 7 days after the formation of an acute subdural hematoma [53]. On the other hand, thrombin is a Janus-faced substrate, as low levels are responsible for protective effects after various neuronal injuries, but excessive upregulation determines in harmful effects [54]. Similar two-way effects were reported in ischemic brains in rats for downstream effects of the PAR1 activation, especially the MAPK/ERK and PI3 K/Akt pathway. Beside cell damaging properties of the MAPK/ERK and PI3 K/Akt system [31, 55], this pathway participates in neuroprotection against transient cerebral ischemia [56, 57]. Apelin-13 is the endogenous ligand of the angiotensin receptor AT1, which is widely expressed in neurons and gliocytes in the central nervous system. Apelin-13 was applied intracerebroventricular 15 min before reperfusion and resulted in a significantly ameliorated neurological deficit, infarct volume, brain edema and reduced TUNEL-positive cells 24 h after stroke [56]. Dexmedetomidine, a highly selective α_2_-adrenergic agonist, in clinical use for the sedation of intensive care and ASDH patients, was administered into the ventricle 30 min before ischemia. The Dexmedetomidine-induced increment of neuron survival 24 h after transient ischemia in the CA1 region and cortex was diminished by the PI3 K inhibitor LY294002 [57]. To influence the thrombin/PAR1 pathway in the right direction it seems to be difficult to determine the right time and localization to prevent deleterious effects of thrombin to the central nervous system. Only early Argatroban treatment 3 h after intracerebral hemorrhage caused a decrease of perihematomal water content for 48 h [58]. 72 h after traumatic brain injury, thrombin levels in brain tissue become upregulated [59]. Systemic continuous Argatroban treatment with intraperitoneal osmotic mini pumps (0.9 mg/h) did not improve functional outcome 24 h after subarachnoid hemorrhage but achieved neurological improvement after 48 and 72 h [18]. Very likely, we have to figure out a so far unknown window of opportunity for blocking PAR1 and consider other participating ligands. Spontaneously triggered cortical spreading depression (CSD) following ASDH could like wisely be triggered by thrombin (but lacking PAR1 activation) and lead to cell death and therefore may contribute to injury development [60]. In the last decades, the t-PA/plasmin system has been carefully observed because tPA is used in thrombolytic therapy for stroke patients [61]. After focal cerebral ischemia [62] and spinal cord injury [63] thrombin and other serin-proteases can lead to post ischemic inflammation and apoptosis, especially via the PAR1 pathway. PAR1 knockout mice displayed reduced signatures of inflammation and astrogliosis, including expression of glial fibrillary acidic protein (GFAP), vimentin, and STAT3, IL-1β and IL-6 signaling 3–30 days after spinal cord injury [64]. These findings demonstrate PAR1 serving as a regulator of the immune cascade. It can be targeted genetically to improve neurobehavioral outcome and to attenuate the excess immune reaction. In our ASDH study we were unable to demonstrate significant effects with pharmaceutical acute local PAR1 inhibition. The therapeutic utility of Thrombin and PAR1 blockage may result from interference with downstream components of thrombin signaling pathways, like seen in model human motor neurons exposed to thrombin [65]. Altogether, thrombin inhibition with Argatroban seems to ameliorate only a small part of the devastating effects occurring via PAR1 activation after ASDH. Another complicating fact is that PAR1 mediates the toxic effects of thrombin in different ways when comparing striatum and cortex [66]. Thrombin induces delayed neuronal injury in organotypic cortico-striatal slice cultures. Thrombin-induced shrinkage of the striatum can be inhibited by Argatroban and the PAR1 antagonist FR171113, whereas thrombin-induced cortical injury is only slightly attenuated by Argatroban [66]. This could explain the sharper and different effects of thrombin and PAR1 blocking in ischemic animal models in contrast to the acute subdural hematoma.

## Conclusion

Our study shows that thrombin may play a contributing role in the development of secondary brain damage after an acute subdural hemorrhage, but not as a primary source of injury when only the acute thrombin-release and PAR1 activation is blocked. There are no significant differences between vehicle solution, local Thrombin-inhibition or PAR1-blocking regarding the damage volume after 14 days. Nonetheless, we demonstrate a pathophysiological mechanism, which is linked to Thrombin and PAR1 during ASDH. In this respect, thrombin may act in concert with other blood-induced pathophysiological processes leading to the devastating effects of ASDH for patients.

## Methods

### Study design, groups, animals and housing

The study consisted of two parts: Direct thrombin inhibition by Argatroban and PAR1-antognism with SCH79797 in a high and low dose. In total 65 rats were randomly assigned to the experimental groups. Six animals had to be excluded due to death before day 14 after ASDH. Finally 59 rats were included in the studies, namely 26 rats for the Argatroban and 33 rats for the SCH79797 trial. All male Sprague–Dawley rats (weighing 295–325 g) were purchased from Charles River Laboratories, Sulzfeld, Germany. All measures were taken to minimize pain and discomfort of animals and experiments including anesthesia were approved by the Animal Ethics Committee of the Landesuntersuchungsamt Rheinland-Pfalz, Germany. Animals were housed in single cages with free access to food and water, at room temperature and humidity of 22 ± 2 °C and 50 ± 5% and at a night day cycle of 12 h under artificial lighting. In all rats middle-artery-blood-pressure (MAP), blood gases, body temperature and cerebral-blood-flow were tightly monitored during anesthesia. All investigators were blinded to treatment during execution and evaluation according to Good Clinical Laboratory Practice.

#### Argatroban study

This experimental series consisted of 10 rats with sham operation, 8 rats receiving autologous subdural blood infusions mixed with 0.9% NaCl (B. Braun, Melsungen, Germany) as a vehicle control group and 8 rats receiving autologous subdural blood infusions mixed with the direct thrombin inhibitor Argatroban (Mitsubishi Pharma, London, UK). Acute pathophysiological parameters, behavioral and histological outcome were assessed on day 14 after ASDH. The animals received 300 µl autologous blood, mixed before injection with either 10 µl vehicle solution or with 600 µg Argatroban mixed with vehicle solution. Sham-operated animals received anesthesia and cannula insertion but no ASDH. The dose of 600 µg Argatroban mixed with 10 µl NaCl 0.9% in 300 µl autologous venous blood was the lowest dose to prevent coagulation of the blood probe for at least 4 h. Venous blood sampling 4 h after subdural injection of Argatroban presented no effect on the activated partial thromboplastin time (data not shown).

#### SCH79797 study

The second experimental series consisted of 8 rats receiving an intracerebroventricular injection of DMSO (vehicle), 10 rats receiving SCH79797 at a low dose (1 µg/10 µl i.c.v.) and 8 rats receiving 10 µl ofSCH79797 at a high dose (5 µg/10 µl i.c.v immediately before ASDH. 7 rats were used as sham-operated controls.

Vehicle and treatment groups received a subdural infusion of 300 µl autologous blood in combination with an i.c.v. injection of either 10 µl vehicle (DMSO), or the PAR1 inhibitor SCH79797 (Axon Medchem, Groningen, NL). Sham animals underwent all operative procedure but received no blood or drug injection. The two SCH79797 concentrations had shown before to be optimal for the amelioration of neurological deficits following surgical brain injury, decreasing mortality and morbidity in epileptogenesis in rats and prevent hydrocephalus after intracerebral hemorrhage in rats [32, 52, 67]. The PAR1-antagonist SCH79797 was administered directly prior ASDH induction into the ventricle via a second burr hole with a 23G cannula (burr hole Ø 1 mm; coordinates; AP = − 1 mm, ML = 1 mm; DV = 3 mm). Since Argatroban mixed to the infused subdural blood (Argatroban study) prevents the immediate release and consequently effect of thrombin on PAR1, SCH79797 was injected immediately before ASDH in order to block the early effects of blood released thrombin and other serine proteases on PAR1 activation similarly. Sham animals underwent the same anesthetic and surgical procedure with cannula insertion and post-interventional surveillance but received no subdural blood infusion or pharmacological treatment.

### Anesthesia and surgical preparation

In both parts of the study the same animal model was applied. As previously described [6, 68] each animal underwent an anesthesia induction by brief isoflurane exposure which was followed by intraperitoneal chloral hydrate injection (36 mg/100 g body weight) and 0.5 mg atropine subcutaneously. Anesthesia was maintained with 36 mg chloral hydrate per hour. Meanwhile we are aware of increasing evidence that the use of chloral hydrate as an anesthetic is inappropriate [69] and we will not advice it in future animal research proposals. Body temperature was kept at 37 °C with a feedback-regulated heating pad and a rectal thermometer (Homeothermic Blanket Unit, Harvard, Kent, UK). All animals received an oral endotracheal intubation and an artificial ventilation (FiO_2_ 28 ± 2%) in order to keep arterial pCO_2_ and pH at physiological levels (Small Animal Ventilator SAR 830, CWE-Inc., USA). After introducing an invasive blood pressure in the tail artery, all animals received a catheter in the right jugular vein to withdraw autologous venous blood for subdural infusion. Samples for blood-gas-analyses (2 × 210 µl) were taken from the tail artery before and after ASDH. The craniotomy was performed 1 mm posterior to the bregma and 1 mm lateral to the midline above the left cortex (diameter 3 mm). Afterwards the dura was opened and a blunt “L”-shaped 23G canula was inserted. The needle was first secured with tissue glue (Histoacryl; B Braun, Melsungen, Germany) and finally fixed with dental cement (Pallavit, Heraeus-Kulzer, Hanau, Germany) to allow an appropriate increase of ICP during subdural blood infusion (300 µl autologous venous blood, 50 µl/min). After induction of the hematoma, the cannula was cut off close to the skull and sealed with tissue glue. A laser Doppler probe (Laserflo BPM 403A, TSI Inc., St. Paul, MN, US) was placed above a 2 × 2 mm thinned out skull area at the ipsilateral hemisphere frontal to bregma. After an equilibration period to maintain stable physiological values, a 10-min baseline period was recorded. For better comparison between animals, the blood flow is given in absolute Laser-Doppler-Units [LDU]. 60 min after ASDH, the recording of the acute parameters MAP and CBF was completed and a final blood gas analysis was performed. All skin incisions were closed with a single-button suture and the animals were returned to their cage.

### Functional testing

For the ASDH with Argatroban treatment (1) a functional testing was implemented, as described before [37]. It has been carried out without pre-injury training on day 14 after injury. The experimentator was blinded to the treatment. In short, neurologic and behavioral testing was performed in dim light in a quiet room. In a first step, the Neurological Deficit Score (NDS) was implemented, ranging from 0 (no deficit) to 9 points (severe deficit), for details see [36]. The NDS was assessed on the basis of sensory, motor and coordination skills and of the general neurological condition of the rat. Deficit points were awarded for state of consciousness, breathing, vision, the whisker movements, hearing, sensing, placing, righting reflex and gait. A Beam Balance Test was used to objectify motor deficits. Each animal was placed on a 2 × 2 cm quadratic 15 cm long wooden beam for maximal 60 s and the time on the beam was assessed. Values in the text and the graphs represent the mean of each group after three trials per animal.

### Histological analysis

All animals were perfusion-fixed by trans-cardiac perfusion with 4% paraformaldehyde (pH 7.4) under anesthesia. Thereafter brains were removed and embedded in paraffin. Coronal brain section. (3 µm) spaced 250 µm apart were stained with hematoxylin–eosin (HE) and lesion volume was determined, as described in detail elsewhere [70]. Damaged tissue included regions with necrotic cells and atrophic areas (i.e. already removed cortical tissue). Pathology which did not relate to the subdural hematoma (damage caused by insertion of the subdural or intraventricular cannula) was noted and the animals with intraparenchymal hematoma were excluded from the study. All analysis was performed blinded to group and treatment.

### Statistics

For statistical analysis we used the program Sigma Plot (SigmaPlot, SigmaStat 2004 for Windows Version 9.01, Systat Software, Erkrath, Germany). All values were expressed as mean values ± standard error of the mean (s.e.m.). Differences with a *P* value < 0.05 were considered statistically significant. The data was analyzed before each test for normal distribution and equal variances, as a condition for the selection of parametric (one-way ANOVA) or non-parametric (Kruskal–Wallis ANOVA on ranks) statistics. A Student–Newman–Keuls (parametric) or Dunn’s test (non-parametric) was used as post hoc test for individual group differences.
